# Persistent High Incidence of Tuberculosis in Immigrants in a Low-Incidence Country

**DOI:** 10.3201/eid0807.010482

**Published:** 2002-07

**Authors:** Troels Lillebaek, Åse B. Andersen, Asger Dirksen, Else Smith, Lene T. Skovgaard, Axel Kok-Jensen

**Affiliations:** * Statens Serum Institut, Copenhagen, Denmark; † Rigshospitalet (National Hospital), Copenhagen, Denmark; ‡ Gentofte University Hospital, Hellerup, Denmark; § University of Copenhagen, Copenhagen, Denmark

**Keywords:** tuberculosis, immigrants, incidence, epidemiology, screening, control

## Abstract

Immigration from areas of high incidence is thought to have fueled the resurgence of tuberculosis (TB) in areas of low incidence. To reduce the risk of disease in low-incidence areas, the main countermeasure has been the screening of immigrants on arrival. This measure is based on the assumption of a prompt decline in the incidence of TB in immigrants during their first few years of residence in a country with low overall incidence. We have documented that this assumption is not true for 619 Somali immigrants reported in Denmark as having TB. The annual incidence of TB declined only gradually during the first 7 years of residence, from an initial 2,000 per 100,000 to 700 per 100,000. The decline was described by an exponential function with a half-time of 5.7 (95% confidence interval 4.0 to 9.7) years. This finding seriously challenges the adequacy of the customary practice of screening solely on arrival.

In most industrialized countries, the annual numbers of cases and deaths caused by tuberculosis (TB) have steadily declined over the past century up to the mid-1980s (1,2) (Figure 1). Since then, an increasing number of TB cases in immigrants has reversed this downward trend in countries that have had substantial levels of immigration from areas with a high prevalence of the disease (1,3,4) (Figure 2). Today, the proportion of immigrants among persons reported as having TB exceeds 50% in several European countries, including Denmark, Israel, the Netherlands, Norway, Sweden, and Switzerland (5). A similar proportion has been predicted for the United States in 2002 (3). In Denmark, the doubling in reported TB cases over the last 15 years has reflected, in large measure, TB in immigrants from Somalia (6), who also account for a sizeable proportion of TB cases in other European countries and North America (7–11).

**Figure 1:**
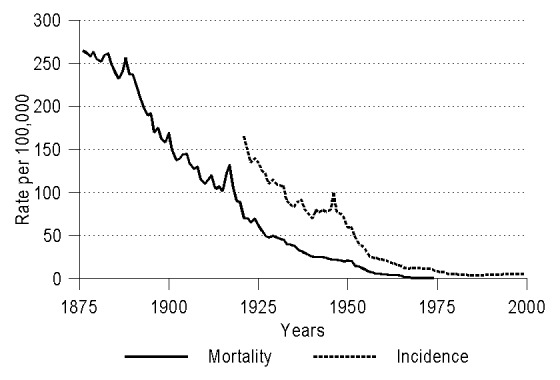
Cases of pulmonary tuberculosis in Denmark over a 125-year period, based on national surveillance information: mortality rates and incidence.

**Figure 2:**
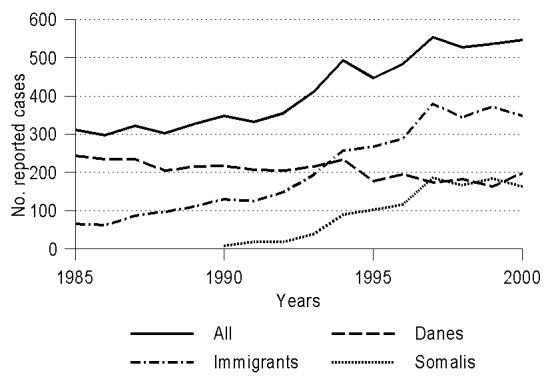
Trends in number of reported cases of tuberculosis in Denmark over the last 15 years, by nationality.

The epidemiologic importance of migration for TB low-incidence countries has been recognized for several years; the main countermeasure has been the implementation of screening programs for immigrants at the time of arrival (4,12). In 1994, 20 of 23 European countries were reported to screen for TB on immigrants’ arrival (4). This measure is based on the fundamental assumption of a prompt decline in the incidence of TB in immigrants from an area of high incidence during their first few years of residence in the country of low incidence. However, only a few studies have actually addressed this question (13–17).

To evaluate the implications for the practice of screening on arrival, we explored the changes with time of residence in the incidence of TB in immigrants from high-incidence areas. Our study focused on the 13,535 Somalis who arrived in Denmark during the 1990s, 901 of whom were subsequently reported as having TB.

## Methods

The study was designed as a nationwide retrospective cohort analysis of surveillance data on all 901 Somalis reported as having TB in Denmark from 1991 to 1999. In Denmark, TB reporting has been mandatory since 1905. Since 1922, all cultures for mycobacteria have been performed at the International Reference Laboratory of Mycobacteriology at Statens Serum Institut in Copenhagen (18). During the study period, this laboratory provided bacteriologic data on all Somalis reported in Denmark as having TB. TB treatment is centralized in departments of respiratory, pediatric, and infectious medicine, which are also responsible for reporting data on all new and recurrent cases of TB by means of a standardized form. These individual reports are collected in a national surveillance register at the Department of Epidemiology at Statens Serum Institut. This department provided information on the annual number of cases, nationality of the patients, and their date of entry into the country. The total number of Somalis in Denmark at the end of each year was taken from the Statistical Yearbooks from 1992 to 2000, published in Copenhagen by Statistics Denmark. The study was approved by the local medical ethics committees (No. 11-087/99) and the Danish Data Protection Agency (No. 2001-41-1018).

The following operational terms were used, adapted to Danish administrative terminology (4). A foreign-born person was any person born outside Denmark, while the term Somali was applied to any person born in Somalia. An immigrant was any foreign-born person legally admitted to Denmark who had already settled in the country or was expected to do so. A refugee was any person who had been granted refugee status by the Danish authorities. An asylum-seeker was any person wishing to be admitted to the country as a refugee but awaiting decision on his or her application for refugee status. An illegal immigrant was defined as any person whose entry, stay, or work in the country was not permitted by the Danish authorities. Illegal immigrants are considered rare in Denmark, and no patient included in the study fell into this category. The word “screening” was used in connection with any interventions performed to discover Mycobacterium tuberculosis infection suitable for early preventive or curative therapy, in a person whose symptoms were not so severe as to cause him or her to seek medical help. The term “medical evaluation” was used in connection with interventions not specifically performed to discover M. tuberculosis infection, e.g., general health examinations.

Over the study period, the number of Somali immigrants in Denmark increased considerably, from 743 in 1991 to 14,856 in 2000 (Table 1). Because of this sustained rise, the annual increase in the number of Somali immigrants was classified as net arrival in Denmark, without taking into account the small numbers who were born, died, or left the country during the study period. For example, at the beginning of 1992 and 1993, respectively, 1,395 and 2,237 Somali immigrants were living in Denmark, corresponding to a net arrival in 1992 of 2,237 – 1,395 = 842 persons (Table 1). To compare changes in numbers from year to year, the observation period for all Somali immigrants included in the study in a given year was totaled as person-observation years (Table 1). The Somali immigrants entered Denmark at various times of the year; therefore, on average each immigrant contributed only a half person-observation year in the calendar year of arrival. For example, in 1992 a total of 1,395 Somali immigrants were already living in Denmark at the beginning of the year. They were observed during the whole calendar year and thus accounted for 1,395 person-observation years. A further 842 Somali immigrants arrived during 1992 and thus accounted for 842/2 = 421 person-observation years, if an even distribution of arrivals is assumed throughout the year. Thus, the total number of Somali immigrants in 1992 was 1,395 + 421 = 1,816 person-observation years (Table 1). Calendar years of arrival and diagnosis were used because exact dates were not available.

**Table 1:** Number of Somalis in Denmark and cases of Somali immigrants reported as having tuberculosis, 1991–2000

Year	*a*	*b*	*c*	*d*	*e*
	Somalis in Denmark^a^ (no.)	Net arrival^b^ (no.) (a_n+1_-a_n_)	Person-observation years yrs (*a* + 1/2*b*)	Reported cases^c^ (no.)	Crude rated (*d* as % of *c*) (95% CI)
1991 and earlier	743	652	1,069	NA	NA
1992	1,395	842	1,816	20	1.1 (0.7 to 1.7)
1993	2,237	1,552	3,013	41	1.4 (1.0 to 1.9)
1994	3,789	1,491	4,535	92	2.0 (1.7 to 2.5)
1995	5,280	1,811	6,186	100	1.6 (1.3 to 2.0)
1996	7,091	2,794	8,488	114	1.3 (1.1 to 1.6)
1997	9,885	2,228	10,999	182	1.7 (1.4 to 1.9)
1998	12,113	1,422	12,824	167	1.3 (1.1 to 1.5)
1999	13,535	1,321	14,196	185	1.3 (1.1 to 1.5)
2000	14,856	NA	NA	NA	NA

^a^Number of Somalis in Denmark by January 1. ^b^Estimated number of Somalis arriving in Denmark in 1 year. ^c^Somalis reported as having tuberculosis (TB). ^d^Crude incidence rate as % of the accumulated number of Somalis in Denmark; NA = not available; 95% CI = 95% confidence interval.

In calculating incidences of TB in relation to duration of residence in Denmark, we gave special attention to Somalis reported as having TB during the period 1995–1999 (Table 2). Each incidence was calculated from the number of Somalis reported as having TB after a given number of years of residence in Denmark, divided by the total number of Somali immigrants who had resided in Denmark for the same number of years. In total, 748 Somalis were reported as having TB from 1995 to 1999 inclusive. Of these, 84 persons were excluded because of lack of information about their exact year of arrival in Denmark, in addition to 45 persons who were known to have arrived in 1991 or earlier (Table 2). Hence, we were able to calculate the risk for developing TB in relation to the average duration of residence in Denmark for 619 Somalis. For example, 158 Somalis were reported as having TB during their second calendar year of residence in Denmark, after an average of 1 year’s residence in Denmark (Table 3). This number represents the sum of the 24 Somalis who arrived in 1994 and were reported in 1995, the 24 who arrived in 1995 and were reported in 1996, the 58 who arrived in 1996 and were reported in 1997, the 34 who arrived in 1997 and were reported in 1998, and the 18 who arrived 1998 and were reported in 1999 (Table 2). The TB incidence was then calculated by dividing by the person-observation years: the 158 Somalis who were diagnosed with TB during their second calendar year of residence in Denmark were found among 9,746 person-observation years for persons who on average had resided in Denmark for 1 year, giving an incidence of 158/9,746 = 1.6% (Table 3).

**Table 2:** Somali immigrants in Denmark reported as having TB, 1995–1999, by year of arrival and diagnosis

Year	Total arrivals	Year of diagnosis (no./yr)					
		1995	1996	1997	1998	1999	Total
1991 and earlier	1,395	8	5	10	8	14	45
1992	842	7	10	7	7	13	44
1993	1,552	30	13	10	18	10	81
1994	1,491	24	22	19	22	20	107
1995	1,811	17	24	29	29	21	120
1996	2,794	-	28	58	31	27	144
1997	2,228	-	-	21	34	19	74
1998	1,422	-	-	-	10	18	28
1999	1,321	-	-	-	-	21	21
Unknown yr of arrival (%)		14	12	28	8	22	84 (11.2)
Total TB^a^ (%)		100	114	182	167	185	748 (100.0)

^a^Total number of Somali immigrants reported as having tuberculosis (TB).

**Table 3:** Risk for tuberculosis (TB) related to duration of residence in Denmark for Somali immigrants, 1995–1999

Average residence^a^ (yrs)	Person-observation years	Reported TB cases		
		no.	Incidence % (no. in % of yrs) (95% CI)^b^	Cumulated annual incidence (%)
1/2^b^	4,788^b^	97	2.0 (1.7 to 2.5)	1.0^b^
1	9,746	158	1.6 (1.4 to 1.9)	2.6
2	9,876	131	1.3 (1.1 to 1.6)	3.9
3	8,490	95	1.1 (0.9 to 1.4)	5.0
4	5,696	63	1.1 (0.9 to 1.4)	6.1
5	3,885	45	1.2 (0.9 to 1.6)	7.3
6	2,394	17	0.7 (0.4 to 1.2)	8.0
7	842	13	1.5 (0.9 to 2.7)	9.5

^a^Average duration of residence in Denmark ^b^Person-observation years and cumulated incidence only “counts half” in year after arrival (see Methods); 95% CI, 95% confidence interval.

For statistical analysis, 95% confidence intervals (CI) were derived from the normal approximation to the binomial distribution (Table 1 and Table 3). The single p-value given in the results was calculated by the chi-square test. If one assumes a Poisson distribution and an exponential decrease in incidence with time, the half-time of the decline in the observed incidences with time of residence was estimated (with 95% CI) by means of SAS statistical software (GENMOD procedure; SAS Institute Inc., Cary, NC).

## Results

### Basic Cohort Data

From 1991 to 1999 in Denmark, 4,147 persons were reported as having TB. Of reported patients, 57.5% (2,386/4,147) were foreign-born, of whom 37.8% (901/2,386) were Somali. For each year, 80%–91% of reported patients were culture positive for M. tuberculosis. Of total culture-positive patients, 74.7% had pulmonary TB with or without extrapulmonary disease, and 25.3% had extrapulmonary disease only. Foreign-born patients had a higher frequency of exclusively extrapulmonary TB than Danish patients (45.6% vs. 16.6%; p<0.001). Of Danish and foreign-born patients with culture-positive pulmonary TB, 55.3% and 26.2%, respectively, had sputum smears positive for acid-fast bacilli.

### Trends in TB Incidence Related to Duration of Residence

**Figure 3:**
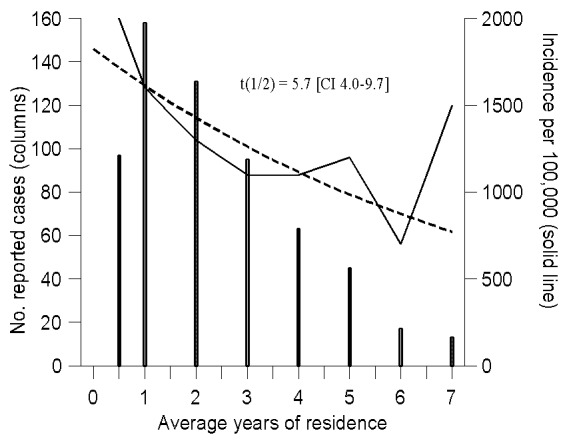
Trend in the incidence of tuberculosis in Somali immigrants in Denmark, by duration of residence. The dotted line indicates the estimated incidence curve and t(1/2) the corresponding half-time, with confidence interval.

The overall annual incidence rate for Somalis remained fairly steady at 1.1%–2.0% (Table 1), but when duration of residence in Denmark was taken into account, the incidence gradually decreased from 2.0% (CI 1.7 to 2.5) during the year of arrival to 0.7% (CI 0.4 to 1.2) during the sixth year of residence (Table 3; Figure 3). The only gradual decrease in incidence rate was described by a simple exponential model with a half-time of 5.7 (CI 4.0 to 9.7) years (Figure 3). Analysis of residuals plotted against duration of residence, year of arrival, year of diagnosis, and person-observation years showed no obvious deviation from a simple exponential model during the first years of residence. During the seventh year of residence, the incidence increased to 1.5% (CI 0.9 to 2.7); however, as seen from the wide CI, this figure is subject to considerable uncertainty. Only 842 Somali immigrants, of whom 13 were reported as having TB, had been living in Denmark long enough to be eligible for observation during their seventh year of residence (Table 3). Overall, 9.5% of all Somalis who arrived in Denmark were diagnosed with TB during their first 7 years of residence (Table 3).

## Discussion

### TB after Arrival in a Low-Incidence Country

Our data show that the initial incidence of TB in Somalis entering Denmark was high, and more importantly, that this high initial incidence declined only gradually, at first in an exponential manner, in the 7 years after arrival. The incidence of TB in Somalis in Denmark is higher than in any other foreign-born population group in the country (19,20) and is comparable with or even higher than the estimated incidence in Somalia (21). During their first 2 years of residence in Denmark, 3.9% of all Somalis were diagnosed with TB, and after 7 years, 9.5% were reported as having TB (Table 3). The exact reasons for the extraordinarily high and only slowly declining incidence of TB in Somalis in Denmark remain unknown, but some theoretical possibilities are discussed in this section.

Epidemiologically, the most important potential impact of excess TB cases due to immigrants from an area of high incidence would be an increase in the rate of transmission in the recipient country of low incidence (22). However, the number of reported TB cases in the Danish-born population has not yet shown any detectable increase in parallel with the increase in the cases in the foreign-born population (Figure 2). Moreover, a nationwide study of M. tuberculosis DNA patterns from 3,320 TB patients in Denmark recently indicated that TB due to transmission among Somalis in Denmark was limited, and transmission between Somalis and Danes was almost nonexistent (6). Recent M. tuberculosis transmission among the Somalis in Denmark cannot explain the high and only gradually declining incidence of the disease after arrival; furthermore, the Somalis in Denmark have not substantially increased their risk for TB infection, as they are diagnosed and treated promptly.

In combination with a high prevalence of dormant M. tuberculosis infection, impairment of the immune system (e.g., as a result of HIV infection) could reactivate latent disease (23,24). Indeed, M. tuberculosis bacteria in immigrants from high-incidence areas may constitute a pool from which active TB could develop. Without taking BCG vaccination status into account, tuberculin skin testing of 300 Somalis in Denmark has indicated that 80%–90% of all adults (16–49 years of age) and 25% of all children were infected with M. tuberculosis at the time of arrival (unpub. data). However, only 1–2 Somali TB patients are found to be HIV positive every year. In addition, four studies in Somalia reported a very low prevalence of HIV infection, even among prostitutes attending a clinic for sexually transmitted diseases in the capital Mogadishu (21,25–27). At present, HIV infection does not seem to play an important role in the development of TB in Somalis.

The mechanisms behind the pattern of incidence of TB in Somalis in Denmark merit further exploration. One of the principal hypotheses is that the immigrant population contains many cases of latent infection with M. tuberculosis that later produce overt disease (28). Factors that could promote this reactivation, which should be identified and examined, include vitamin deficiencies, genetic constitution, and immune defects. In the preantibiotic era, the risk of reactivation of TB after recovery was extraordinarily high: annual relapse rates were 4.4% during the first 5 years and 1.6% during the next 5 years (2). The situation in Somalia and the refugee camps from which the immigrants have come may resemble the preantibiotic era in the high number of relapses and reactivations now being observed. In addition, many cases of TB may not have been identified in the refugee camps, or if they were diagnosed, patients may not have received proper treatment because of lack of resources (16). The discovery of such cases during screening on arrival in Denmark could explain the high initial incidence and why this rate could exceed the estimated rate in Somalia, where cases may remain unrecorded (21).

### Implications for Policy of On-Arrival–Only Screening

Only a few studies have described the trend in TB incidence in immigrants over the years after their arrival from an area of high incidence (13–17). Three of these studies were restricted to immigrants arriving from Asia (14–16), and two covered only a short period of observation (16,17). The general finding was of a prompt decline in incidence during the first few years of residence in the receiving country, although two studies reported an increased TB risk many years after arrival, as we observed for the Somalis in Denmark (13,16). The observation of a prompt decline in incidence has had a major influence on the countermeasures taken to prevent and control the disease in low-incidence countries. Nearly all low-incidence countries have implemented programs in which immigrants are screened only at the time of arrival (4,12): in 1994 20 of 23 European countries followed this practice (4).

In Denmark, all refugees and asylum seekers are encouraged to have a general medical evaluation (not specific for TB) only at the time of arrival in the country. Those who do not arrive as refugees or asylum seekers do not undergo systematic medical evaluations but are entitled to contact the free public health-care system on their own initiative. After the initial medical evaluation, the immigrants, refugees, and asylum seekers in Denmark, as in most other low-incidence countries, are covered by the national TB program, which is based on passive case-finding and treatment of active cases, combined with contact tracing (29). This program involves chest x-ray examination if pulmonary symptoms persist for >6 weeks, examination for M. tuberculosis if chest x-ray is suggestive of TB, examination by chest x-ray every 6 months for 3 years in tuberculin-positive subjects who have had recent exposure to a smear-positive TB patient, examination for M. tuberculosis from extrapulmonary sites if symptoms indicate TB, free four-drug short-course treatment regimens for TB patients, and preventive chemotherapy only for children <7 years of age whose tuberculin skin test is positive (29).

The gradually declining incidence in the years after arrival observed for Somalis in Denmark, persons of different nationalities in the United States (13), and Asians in Canada (16) seriously challenges the adequacy of the policy of screening only on arrival. National TB programs in low-incidence countries should be expanded to include surveillance of trends in the incidence of TB in specific immigrant populations during subsequent years as well. If a gradual decline similar to that in the Somalis in Denmark and Asians in Canada is observed, the present policy of screening only on arrival needs to be revised and refocused. Such revision would probably include as an important feature the institution of voluntary regular health examinations, at reasonable intervals after arrival, for specifically identified high-risk immigrant groups, as the risk may persist for many years. Intervention needs to be an ongoing process that includes both latent M. tuberculosis infection as well as active TB.

Another way of preventing TB in high-risk groups such as the Somalis in Denmark could be preventive chemotherapy, i.e., treatment of persons with subclinical M. tuberculosis infection. Several controlled studies have documented the effectiveness of such a strategy in preventing progression to TB or reactivation of disease on an individual basis (30), but the effectiveness of preventive chemotherapy administered to population groups needs further evaluation (22,31). The compliance of participants is crucial for obtaining satisfactory results (30). For instance, a large meta-analysis showed that only 60.5% of 1,084,760 persons completed preventive therapy (32). If preventive therapy is used indiscriminately, a large number of infected persons would have to be treated to prevent the occurrence of a single case of TB (30), and all those treated would be at risk of side effects from the medication (32). However, preventive therapy may decrease illness for the 9.5% of Somalis who have TB during the first 7 years of residence in Denmark, if the medication is efficiently distributed to the Somalis with latent infection.

### Focused Intervention: Key to Future Control?

As TB declines in low-incidence countries, M. tuberculosis transmission is markedly reduced, and most cases arise in persons who have previously been infected (3). Most cases of TB infection have been acquired in the same country, as has been observed for most older Danish-born TB patients, or have been acquired in another country where TB is still actively transmitted and subsequently been imported, as observed in the Somalis in Denmark (6). Thus, in low-incidence countries TB has increasingly come to be a disease of specific subgroups of the population (22). This trend provides an opportunity for focused intervention, the success of which will depend on correctly identifying the population groups at risk. Because of the considerable geographic variations in TB in immigrants from different countries and different trends in incidence after arrival in various host countries, approaches to controlling and preventing TB should be tailored to the specific foreign-born populations at risk. Control and elimination strategies should be focused on diminishing the incidence and prevalence of latent infection to reduce the pool of TB infection from which future cases of TB will emanate. This goal can be accomplished by two approaches: first, to reduce the incidence of new TB infection and thereby limit the growth of the pool and second, to reduce its prevalence (33). To arrest the chain of transmission, the risk of new generations becoming infected must be minimized by the early identification and curative treatment of newly emerging infective sources. Furthermore, newly infected persons must be prevented from progressing to overt disease; this approach reduces the number of cases caused by recent transmission (3). Our study also underlines the importance of transition from latent infection to active disease. If we seek to control the rates of TB in immigrants arriving from areas of high incidence, the success of our control measures will increasingly depend on reducing the impact of TB in immigrants by arresting the transition from latent to active disease. However, the global perspective of TB should also be kept in mind: the impact of disease falls principally on developing nations, where 95% of all cases and 98% of deaths due to TB occur (34). Intervention in such high-incidence areas, in addition to intervention in the low-incidence countries, is still crucial for the elimination of TB.
